# *R*_0_: Host Longevity Matters

**DOI:** 10.1007/s10441-018-9315-1

**Published:** 2018-02-19

**Authors:** L. M. Viljoen, L. Hemerik, J. Molenaar

**Affiliations:** 1 Department of Mathematics and Applied Mathematics, North West University, Potchefstroom, North West South Africa; 2 Biometris, Department of Mathematical and Statistical Methods, Wageningen University, Wageningen, The Netherlands

**Keywords:** Epidemiology, *R*_0_, Host longevity, Duration of infection, Fitness strategy, milker–killer dilemma

## Abstract

The basic reproduction ratio, *R*_0_, is a fundamental concept in epidemiology. It is defined as the total number of secondary infections brought on by a single primary infection, in a totally susceptible population. The value of *R*_0_ indicates whether a starting epidemic reaches a considerable part of the population and causes a lot of damage, or whether it remains restricted to a relatively small number of individuals. To calculate *R*_0_ one has to evaluate an integral that ranges over the duration of the infection of the host. This duration is, of course, limited by remaining host longevity. So, *R*_0_ depends on remaining host longevity and in this paper we show that for long-lived hosts this aspect may not be ignored for long-lasting infections. We investigate in particular how this epidemiological measure of pathogen fitness depends on host longevity. For our analyses we adopt and combine a generic within- and between-host model from the literature. To find the optimal strategy for a pathogen from an evolutionary point of view, we focus on the indicator $R_0^{{opt}}$, i.e., the optimum of *R*_0_ as a function of its replication and mutation rates. These are the within-host parameters that the pathogen has at its disposal to optimize its strategy. We show that $R_0^{{opt}}$ is highly influenced by remaining host longevity in combination with the contact rate between hosts in a susceptible population. In addition, these two parameters determine whether a killer-like or a milker-like strategy is optimal for a given pathogen. In the killer-like strategy the pathogen has a high rate of reproduction within the host in a short time span causing a relatively short disease, whereas in the milker-like strategy the pathogen multiplies relatively slowly, producing a continuous small amount of offspring over time with a small effect on host health. The present research allows for the determination of a bifurcation line in the plane of host longevity versus contact rate that forms the boundary between the milker-like and killer-like regions. This plot shows that for short remaining host longevities the killer-like strategy is optimal, whereas for very long remaining host longevities the milker-like strategy is advantageous. For in-between values of host longevity, the contact rate determines which of both strategies is optimal.

**Electronic supplementary material:**

The online version of this article (10.1007/s10441-018-9315-1) contains supplementary material, which is available to authorized users.

## Introduction

In general, successful parasites engage in a dynamic co-evolutionary interaction with their host population. As such, they do not eradicate their hosts. Instead, they live in some kind of stable hostility, resulting in an arms race between parasite and host (Haraguchi and Sasaki 1996). For vertebrate hosts and their obligatory directly transmitted pathogenic microbes this arms race is taking place between the hosts’ immune system on the one hand, and the complex genetic systems that the pathogens develop on the other hand. The main tools pathogens have at their disposal are antigenic diversification and variation in replication rates. These two mechanisms are essential to the pathogen’s continued struggle to evade the host’s immune system that is adapting to control the proliferation of infectious organisms (Deitsch et al. 2009).

The clash of the above-mentioned antigenic players can lead to different optimal strategies for the pathogen. To achieve ecological success (i.e., persistence within a host population), pathogens require mechanisms both for survival within hosts and transmission between hosts. Deitsch et al. (2009) consider some of these strategies and in particular pay attention to the mechanisms of antigenic variation adopted by pathogens to avoid eradication by the hosts’ immune system in order to maintain persistent infections. Herewith, they ensure the feasibility of their transmission to new hosts. Two main strategies combining within- and between-host dynamics are the so-called ‘milker-like’ and ‘killer-like’ strategies (van Baalen and Sabelis 1995). The ‘milker-like’ strategy relates to a pathogen replicating slowly within the host in an attempt to damage the host minimally. In contrast, the ‘killer-like’ strategy refers to a pathogen replicating very quickly and making the host ill. The latter strategy puts all emphasis on a high release rate of pathogens.

In most epidemiological models, the main tool used to identify the ecological success of a pathogen is the basic reproduction ratio, *R*_0_ (Diekmann et al. 1990; Heesterbeek 2002). This measure of pathogen fitness is widely applied, because it provides information regardless of the specific characteristics of the infection under consideration, such as prevalence, virulence change, host switch, and control escape. *R*_0_ is defined as the expected number of secondary infections arising from a single individual during its entire infectious period, in a population of susceptible hosts. See, e.g., Anderson and May (1995), Diekmann and Heesterbeek (2000), Keeling and Grenfell (2000) and Heffernan et al. (2005). An important conclusion from their work is that if $R_0 < 1$, the infection does not disappear immediately, but subsequent generations of infected organisms are smaller and smaller in size. Alternatively, $R_0 > 1$ indicates a possible outbreak of the disease (van den Driessche and Watmough 2002).

Traditionally, *R*_0_ was incorporated in studies that relate to between-host dynamics. However, to account for the complex genetic systems that pathogens have developed, nowadays much attention is given to within-host competition between strains of a given pathogen, as opposed to the implicit assumption that hosts are exploited by a single clone of pathogens (see e.g., Anderson and May 1979; Ewald 1983). When combined with the more traditional population-level approach, this relatively new approach to infectious disease modelling is very promising to gain better understanding of the arms race described above. Consequently, when using systems in which within- and between host processes are modelled simultaneously, *R*_0_ depends on a combination of host-specific and pathogen-related characteristics. One of these characteristics is the remaining longevity of the host.

Increased computational capabilities brought on by technological advances has allowed to consider mathematical models that combine within- and between-host interactions as opposed to viewing the two systems in isolation (see e.g., Bhattacharya et al. 2014; Numfor et al. 2014). Identifying and analysing both host- and pathogen-specific characteristics that influence the spread of an infection in a host-population, is nowadays a topic of intense research. Traditionally, in this field much emphasis has been placed on factors such as force of infection, transmission probabilities, contact neighbourhoods, etc. Of late, the different transmission mechanisms also receive more and more attention. E.g., Rohani et al. (2009), and Brooks-Pollock et al. (2014) consider the impact of environmental transmission mechanisms on control efforts related to various microorganisms. Heinzmann et al. (2011) utilise density based models to investigate environmental factors and evaluate intervention programs. Alexander et al. (2009) evaluate various treatment strategies for influenza infection—with *R*_0_ as threshold parameter - and the optimal timing of treatment campaigns.

In the basic definition of *R*_0_ given in Eq. (9) below, the integral is taken over the entire infectious period. See, e.g., Diekmann and Heesterbeek (2000) and Anderson and May (1995). Although the upper boundary in this integral is taken as $\infty$, the effective length of integration is determined by the transmission rate *q*(*t*) in the integrand, which is defined in Eq. (4). This transmission rate vanishes as soon as the infection dies out. This may be caused by recovery but also by the death of the host. The first is automatically included via (4), but the latter must be introduced by setting the upper boundary at a finite value. We remark that in simulation studies the upper boundary in the integral must always be set at a finite duration, as done by, e.g., Lange and Ferguson (2009), since numerically the integration cannot exceed to $\infty$. However, as we show in this paper, the value used for the upper boundary may strongly determine the model predictions and can be decisive whether the milker-like or the killer-like strategy is optimal. The reason for this is not hard to understand: a milker-like strategy can only last for the specific chosen value of host longevity. In the literature some studies consider host longevity. Examples are a study of the evolution of virulence under the trade-off between transmission probability and host longevity (Sigmund et al. 2002), and a study of the effects of shortened host life span on the evolution of parasite life history and virulence (Nidelet et al. 2009).

The specific relationship between *R*_0_, as a measure of pathogen fitness in a host population, and host longevity has not yet received attention. Therefore, we explicitly study the effect of a finite host longevity on *R*_0_. To investigate this relationship we take an existing model from the literature with no extra mortality due to the infection. In our simulations we use dimensionless versions of these models and by applying numerical procedures that are computationally very efficient, we are able to perform extensive parameter scans. Taking the (remaining) host longevity variable enables us to draw new conclusions about how host longevity shapes the maximum pathogen fitness strategies.

## Methods

We first discuss the modelling concepts underlying the present study. The model used is taken from Lange and Ferguson (2009). The numerical implementation is optimized to allow for many and long computer simulations. We started with replicating their results (see Fig. A1 in Appendix A) to check for computational correctness. Although the modelling principles used in our analysis are thus not new, we prefer to present them in this paper in a self-contained way to avoid unnecessary reference to the literature.

In the next section we describe the modelling of the dynamical evolution of an infection in a host. This *within-host model* represents how in an infected host the number of pathogens evolves in time. Because we allow mutations, the model may deal with a variable number of pathogen strains, each with its own dynamics, depending on their interactions with the immune system of the host and the availability of resources.

Next, we describe the model that represents how an infection spreads in a population. This *between-host model* incorporates the effect of one infected host in a direct and an indirect way: it may infect its direct neighbours directly, and it may infect its neighbours via other neighbours that were already infected by this host at an earlier time. To that end, we include in an averaged way the structure of the relational network of the population.

Eventually we come to the essence of the paper by coupling the two models and considering the *R*_0_ of the coupled system. *R*_0_ depends on host longevity and it is this dependency that gives rise to remarkable insights, as shown in the Sect. 3.

### Within-Host Model

To describe how a pathogen replicates within a host and how the immune system of the host reacts in response to the presence of the pathogen, we use a model with a variable number of strains. The idea is as follows:

We start the invasion in the host with one specific strain. This strain replicates and builds up a viral load, and at the same time evokes an immune response. When replicating a mutation may take place with probability $\mu$. Most mutations will not lead to a new strain type. The fraction of mutations that is ’successful’ and generates a new strain type is equal to parameter $\delta$. In the simulations this process of replication and mutation is modelled using a Poisson process. After the first successful mutation a second strain type comes into existence, with its own dynamics. These two strains may mutate, but may also die and disappear from the system. The consequence is that the number of strains *n* is stochastically varying. The immune system of the host may adapt to recognize new strains. The specific mortality rate of strain *i* due to the immune system of the host is weighted by the difference between between the new strain and the strains that are already present. As a quantative measure for this difference we use the so-called Hamming distance, which is explained below.

The state variables in the within-host model are: $V_i$, the number of pathogens of strain *i*, also referred to as pathogen load *i* ; $X_i$, the adaptive immunity of the host specific to strain *i*; and *C*, the resource level, i.e., the number of target cells available for any strain to multiply.

The dynamics of pathogen load $V_i$ is governed by the following differential equation:

1 $$\begin{aligned} \frac{{d}V_i}{{d}t}&\equiv {} \hbox {[pathogen growth] }- \hbox {[natural death]} -\hbox {[death due to acquired host immunity]} \nonumber \\&= {} (1 - \mu )\,\rho \, V_i\, \frac{\nu _1 C }{ \nu + \nu _1 C} - \psi \, V_i - \sigma \, V_i\, \sum _{k=1}^{n}\,y(\varrho _{ik})\,X_k . \end{aligned}$$

where $\mu$ is the probability that a mutation occurs, $\rho$ the replication rate of the pathogen, $\nu _1$ a conversion factor: it is the number of pathogens that may stem from one unit of resource, and $\nu$ the total viral load in the host, $\nu \equiv \sum _{i=1}^{n}{V_i}$. Equation (1) links pathogen replication to two inhibiting mechanisms: host immunity and resource limitation. Pathogen growth is restricted via a Monod function that shows saturation behaviour if $\nu \gg \nu _1 C$. This term links the pathogen load $V_i$ to the resource level *C*. The last two terms in (1) represent the clearance of pathogen: $\psi \, V_i$ gives the natural clearance of pathogens from a host, independent of any immune response, while the term with $\sigma$ represents the decrease in pathogen load due to immunity acquired by the host. Here, the latter term includes cross immunity via $\sum _{k=1}^{n}{y(\varrho _{ik})\,X_k},$ which models via the Hamming distance $\varrho _{ik}$ (see below) how *similar* strain *i* is to any other strain *k* that is currently in circulation.

In the computer simulations a strain is represented by a string of 5 elements (representing loci) and each element may attain 3 different values (representing alleles). Altogether this allows for 243 possible, different strains.

We use the Hamming distance $\varrho _{ik}$ as a metric that relates similarities between antigenic variants, where $\varrho _{ik}$ is defined as the fraction of loci at which strains *i* and *k* differ. The relative Hamming metric is widely used in the literature to measure antigenic variation. For other possible choices, see e.g. Cai et al. (2012), Plotkin et al. (2002), and Neher et al. (2016). The degree of cross-immunity is incorporated through the function $y(\varrho _{ik}),$ where $y(\varrho _{ik}) = 1 - (1 - \chi )\varrho _{ik}$ if $\varrho _{ik} \le 1/(1 - \chi )$, and $y(\varrho _{ik}) = 0$ otherwise. Parameter $\chi$ thus regulates the degree of cross-immunity with $\chi =0$ in case of total cross immunity. Increasing $\chi$ values correspond with less and less cross immunity.

The dynamics of the adaptive immune response $X_i$ is governed by

2 $$\begin{aligned} \frac{{d}X_i}{{d}t}& \equiv [\hbox{decline of immunity}] + [\hbox {acquisition of immunity}]\\& = \xi \, (x_0 - X_i) + \zeta \ X_i \, \frac{V_i}{\eta + V_i }. \end{aligned}$$

In (2), the first term describes the decline of immunity with $x_0$ the minimum immune level. Note that in general $X_i > x_0$. The acquisition of immunity depends on the load of strain *i* via a Monod function.

The dynamics of the resource level *C* is modelled as:

3 $$\begin{aligned} \frac{{d}C}{{d}t}&\equiv {} \hbox {[limited exponential growth]} - \hbox {[use by present pathogens]} \nonumber \\&= {} \kappa \, (C_0 - C) - \frac{\rho }{\nu _1} \sum _{i=1}^{n}{ V_i\, \frac{\nu _1 C}{ \nu + \nu _1 C}} . \end{aligned}$$

In any host, resource is being replenished at a rate $\kappa$, with $C_0$ a maximum value for *C*, chosen to represent a realistic number of target cells. Note that in general $C < C_0$.

As in an infected host each pathogen strain *i* grows at a rate proportional to $V_i\, (\nu _1 C / ( \nu + \nu _1 C)$,) the resource is depleted proportionally to the sum of all these growth terms.

Because we allow the generation of new strains, the number of pathogen strains is variable, and so is the number of differential equations in our model. If at some moment in time we have *n* different strains, the model consists at that moment of $2n + 1$ differential equations. We solve differential equations (1), (2), and (3) to obtain the time evolution of $V_i(t), X_i(t), i = 1, \ldots , n$, and *C*(*t*) until a new successful mutation takes place. At that moment we replace *n* by $n+1$ and extend the set of model equations with two new equations. It may also happen that we have to decrease *n* with 1, namely when one of the pathogen strains goes extinct. This happens when $V_i \le \nu _0$.

The model in Eqs. (1–3) yields state variables that vary greatly in magnitude, with total pathogen load reaching levels of $\nu \approx 10^{11}$, while adaptive immunity is saturated at $\eta = 10^5$. In contrast with these high values, most parameter values (see Table 1) are of order 1. To allow us to investigate the relationships between parameters and the influence of specific parameters on the dynamics of the system, it is therefore essential to rewrite the model in dimensionless form. For the non-dimensional version we refer to Appendix B. The descriptions of the parameters and state variables of the within-host model are listed in Table 1.

**Table 1:** Parameters and variables of the within host model

	Description	Default value
*Parameter*		
$\rho$	Replication rate of pathogen	$\rho \in [3,8]$
$\delta$	fraction of mutations that are ‘successful’	$\delta \in \left[ 10^{-9},10^{-3}\right]$
$\mu$	Probability that a replication leads to a mutation	0.1
$\nu _0$	Initial and minimum pathogen load	10
$\nu _1$	Conversion factor from resource to pathogens	$10^3$
$C_0$	Initial/maximum resource level	$10^8$
$\psi$	Clearance rate of pathogen	0.25/day
$\sigma$	Immune-related clearance rate	$10^{-3}$/day
$\eta$	Critical load of saturated immune response	$10^{5}$
$\xi$	Decline of immunity	0.3/day
$x_0$	Initial/minimum immunity	1
$\zeta$	Growth of immunity	0.8/day
$\kappa$	Replenishment of resources	1/day
$\chi$	Degree of cross-immunity	0.6
*Variable*		
$V_i (t)$	Number of pathogens of strain *i*	
$X_i (t)$	Adaptive immunity against strain *i*	
*C*(*t*)	Number of target cells available for a strain to multiply	
$\nu (t)$	Total pathogen load	$\sum _{i=1}^{n}{V_i(t)}$

### Between-Host Model

**Fig. 1:**
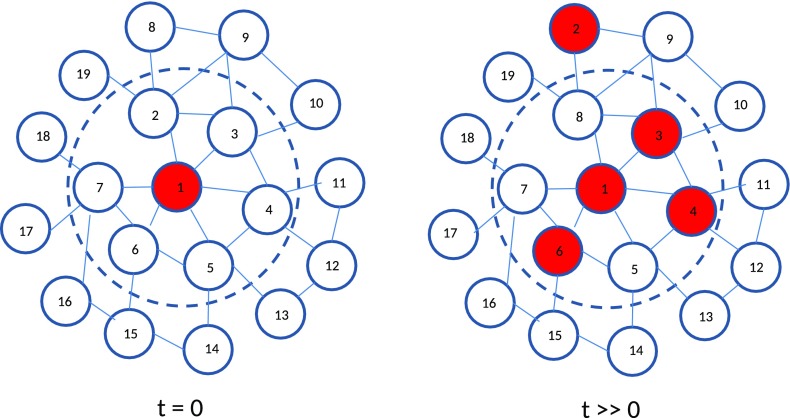
Left: initial network of a susceptible population with one infected host (node 1). This infected host is surrounded by 6 direct neighbours (within the dashed circle), so for the local subnetwork we have $N=7$. Right: the same part of the network after some time. Some of the neighbours of node 1 are now also infected. The infection of node 4 could be caused by its link with node 1 (direct infection), but could also stem from its contact with node 3 (secondary infection). Note that some of the infected neighbours in the local neighbourhood of node 1 may be in the meantime replaced by new susceptible ones. In the present case this happened to node 2, which has exchanged position with node 8

As for the transmission of the pathogen between hosts, our model is based on the use of a network structure as investigated in Keeling (1999). Let us focus on one initially infected host connected to $N-1$ susceptible, but still healthy neighbours. As an example, a sub-network with $N=7$ nodes is sketched in Fig. 1.

In this part of the network the number *S* of susceptible nodes is thus initially $S(0) = N-1$. *S*(*t*) will decrease in time, because the infected host may transfer its infection to its neighbours. The transmission rate *q* will depend on the pathogen load in the infected host, and thus on its age. It is modelled as

4 $$\begin{aligned} q(t) = \beta \left[ 1 - {\text {exp}}\left( -\frac{\nu (t)}{\nu _T}\right) \right] . \end{aligned}$$

So, *q*(*t*) depends on the between-host parameter $\beta$ (transmission rate) and the dynamic within-host total pathogen load, $\nu (t)$. Parameter $\nu _T$ is the infectiousness threshold. If $\nu \gg \nu _T$, *q* will no longer depend on $\nu$ and converge to $\beta$. The transmission rate $\beta$ is defined as $\beta \equiv \alpha \,\gamma /N$ with $\alpha$ the contact rate and $\gamma$ the transmission probability per contact $(\gamma \in (0,1))$.

It remains to model how *S*(*t*) evolves in time. This is done in an averaging way. In our sub-network the total number *N* of nodes is conserved and the number *I* of infected nodes and the number *S* of susceptible nodes sum up to the total number of nodes, $I+S = N$. At the initial time $t=0$, we can simply write

5 $$\begin{aligned} \frac{{d}I}{{d}t}(t) = q(t)\,S(t), \end{aligned}$$

because only direct infections may occur. However, at times $t>0$ the infestation of a susceptible could not only be direct, but also secondary, i.e., due to other neighbours that in the meantime got infected by the originally infected host. When these neighbours were infested $\tau < t$ times ago, this happened with a chance proportional to the transmission rate $q(t - \tau )$ at that moment. This leads to the extended integro-differential equation

6 $$\begin{aligned} \frac{{d}I}{{d}t}(t) = S(t) \left[ q(t) +\phi \,\int _0^t \frac{{d}I}{{d}t}(\tau )\,q(t-\tau ) \, {d}\tau \right] . \end{aligned}$$

Here, $\phi$ defines the cliquishness of the network, i.e. the proportion of neighbours of a node who are neighbours of each other. The integral represents the secondary infections caused by new infectives up to time *t* and takes into account the changing transmission rates resulting from time dependent pathogen loads.

Using that ${d}I/{d}t = - {d}S/{d}t$, we can rewrite this in the form

7 $$\begin{aligned} \frac{{d}S}{{d}t}(t) = - S(t) \left[ q(t) - \phi \,\left( q * \frac{{d}S}{{d}t}\right) \right] \,, \end{aligned}$$

with the convolution of two functions *f*(*t*) and *g*(*t*) defined in the usual way as $(f * g)(t) \equiv \int _0^t f(\tau )\,g(t - \tau )\,{d}\tau$.

As a last modelling step we assume that the infected nodes in our sub network are replaced at a rate $\omega$ by susceptible nodes from the environment in a constantly ongoing exchange process. That process is in a quasi- steady state, so that *N* remains conserved. In (7) this effect is incorporated by replacing *dS* / *dt* with ${d}S/{d}t - \omega \, I$. Eventually we then arrive at

8 $$\begin{aligned} \frac{{d}S}{{d}t} = \omega \,(N-S) - S\,\left[ q + \phi \, q * \left( \omega (N-S) - \frac{{d}S}{{d}t}\right) \right] . \end{aligned}$$

The parameters of the between-host model, together with descriptions, are listed in Table 2.

**Table 2:** Parameters and variables of the between host model

	Description	Default value
*Parameter*		
$\alpha$	Contact rate between hosts	$\alpha \in [0,10]$
$\omega$	Replacement rate of hosts	0.001
$\gamma$	Probability of transmission	0.2
$\beta$	Transmission rate	$\beta = \alpha \gamma / N$
$\phi$	Cliquishness	0.75
$\nu _T$	Infectiousness threshold	$10^8$
$\varpi$	Expected maximum pathogen load	$10^{11}$
*N*	Neighbourhood size	20
*Variable*		
*S*	Number of susceptible hosts	
*q*(*t*)	Transmission rate between hosts	$q = \beta \left( 1 - e^{-\nu (t)/\nu _T}\right)$

### Basic Reproduction Ratio *R*_0_

The basic reproduction ratio, *R*_0_, is a fundamental concept in epidemiology. It is defined as the total number of secondary infections brought on by a single primary infection, in a virgin, susceptible population. The value of *R*_0_ indicates whether a single infection may lead to an epidemic or will probably die out soon. According to Diekmann and Heesterbeek (2000), *R*_0_ is given by the integral

9 $$\begin{aligned} R_0 = \int _0^{\infty } S(t)\,q(t)\,{d}t. \end{aligned}$$

Here, *q*(*t*), defined in (4), is the transmission rate from the infected host to the surrounding susceptibles. This rate depends on the pathogen load in the infected host, which is obtained by evaluating the within-host model. *S*(*t*) is the number of susceptibles in the neighbourhood of the primary infective. It is obtained as a solution of the between-host model in (7) or (8).

The upper boundary in the integral in (9) is $\infty$. In computer simulations one can only work with a finite upper boundary. In Lange and Ferguson (2009) the authors interpret the upper bound as the maximum duration of infection and they take as a cut off point a fixed value of 2 years. This value is in many practical cases sufficiently long. In general however, this may introduce inaccurate results, since in practice the maximum duration of an infection is limited by the host’s (remaining) longevity. That is why we take in this paper a finite upper boundary $D_{{max}}$ and use the definition

10 $$\begin{aligned} R_0 = \int _0^{D_{{max}}} S(t)\,q(t)\,{d}t. \end{aligned}$$

It is clear that *S*(*t*) and *q*(*t*), and thus also *R*_0_, depend in quite a complex way on the model parameters summarized in Tables 1 and 2. If our only goal would be to show how *R*_0_ depends on $D_{{max}}$, it would suffice to fix all parameter values, and to vary $D_{{max}}$. However, our aim is more intricate: we want to look at strategies that pathogens may develop under different circumstances. For this we assume that the pathogen attempts to optimize *R*_0_ in the course of time. For this purpose, the pathogen has two parameters at its disposal, namely the replication rate $\rho$, and the fraction of successful mutations $\delta$. To find the optimal strategy that a pathogen will develop in an evolutionary process, we optimize *R*_0_ with respect to these two within-host parameters. As for the between-host dynamics, the dominant parameter is the contact rate $\alpha$. This parameter is not under control of the pathogen. Consequently, we consider $R_0 = R_0(\rho , \delta ;\alpha , D_{{max}})$ and we are in particular interested in the dependence of *R*_0_ on the contact rate $\alpha$ and host longevity $D_{{max}}$. That’s why we optimize over $\rho$ and $\delta$:

11 $$\begin{aligned} R_0^{{opt}} (\alpha , D_{{max}}) = \max \limits _{(\rho , \delta )} \, R_0(\rho ,\delta ;\alpha , D_{{max}}). \end{aligned}$$

$R_0^{{opt}}$ measures the ‘success’ of a pathogen when adjusting its replication and mutation parameters $\rho$ and $\delta$.

The optimization of Eq. (11) is done by calculating $R_0(\rho ,\delta ;\alpha , D_{{max}})$ on a rectangular grid in the ($\rho ,\delta$) plane and the optimum is simply found by comparison of all grid points.

Solving the within-host system of the system of differential equations is done numerically by using a fourth order Runge Kutta method. Subsequently, Eq. (11) is maximized using the well-known optimisation method of Levenberg and Marquardt.

## Results

We combined and implemented the within- and between-host models in Eqs. (1–8) and, as a check, first recalculated the results as reported in Lange and Ferguson (2009). We found the same results; they are given in Appendix A and B. Next, we focussed at finding how the evolutionary optimal $R_0^{{opt}}$ depends on contact rate $\alpha$ between hosts and host longevity $D_{{max}}$.

To ensure realistic within-host model dynamics that allows for an infection to become endemic in a host, it suffices to use the ranges $\rho \in [3,8]$ and $\delta \in [10^{-9},10^{-3}]$. For the contact rate we take as a realistic range $\alpha \in [0,10]$. The maximum of *R*_0_ over ($\rho$, $\delta$) for fixed values of $\alpha$ and $D_{{max}}$ is found by calculating *R*_0_ on a grid in the ($\rho$, $\delta$)-plane. For this we need to evaluate the within- and between-host models for many ($\rho$, $\delta$)-pairs. This time consuming procedure yields a so-called fitness landscape. An example is given in Fig. A1 of Appendix A, where we not only present *R*_0_, but also the cumulative pathogen load and the duration of the infection, as functions of ($\rho$, $\delta$).

**Fig. 2:**
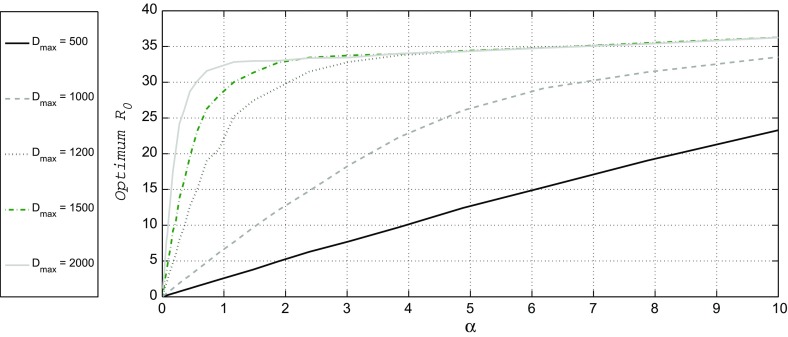
$R_0^{{opt}}$ as a function of the contact rate between hosts ($\alpha$), for various values of host longevity $D_{{max}}$

**Fig. 3:**
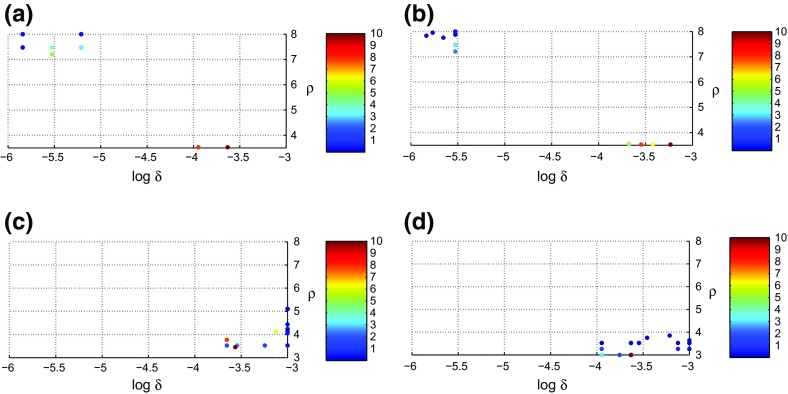
Position of $R_0^{{opt}}$ in the $(\rho , \delta$) plane, with $\rho$ the replication rate of pathogen and $\delta$ the mutation rate of pathogen within the host. This position depends on the between-host contact rate $\alpha$. The values of $\alpha \in (0,10)$ are indicated with colours and colour bars. The position of $R_0^{{opt}}$ also depends on remaining host longevity $D_{{max}}$. Results are shown for 4 values of $D_{{max}}$: **a** $D_{{max}} = 500$, **b** $D_{{max}} = 1000$, **c** $D_{{max}} = 1500$, and **d** $D_{{max}} = 2000$

In Fig. 2 we show $R_0^{{opt}}$ as a function of $\alpha$, for the values $D_{{max}} = 500$, 1000, 1200, 1500, and 2000 days. The results in Fig. 2 exhibit remarkable behaviour. If $D_{{max}}$ is relatively short ($D_{{max}} = 500$) days, the dependence of $R_0^{{opt}}$ on $\alpha$ is nearly linear over the full interval that we tested. This implies that, when the contact rate increases, the success of the pathogen in spreading itself in a host population goes up in a way proportional to the number of contacts, as is to be expected.

**Fig. 4:**
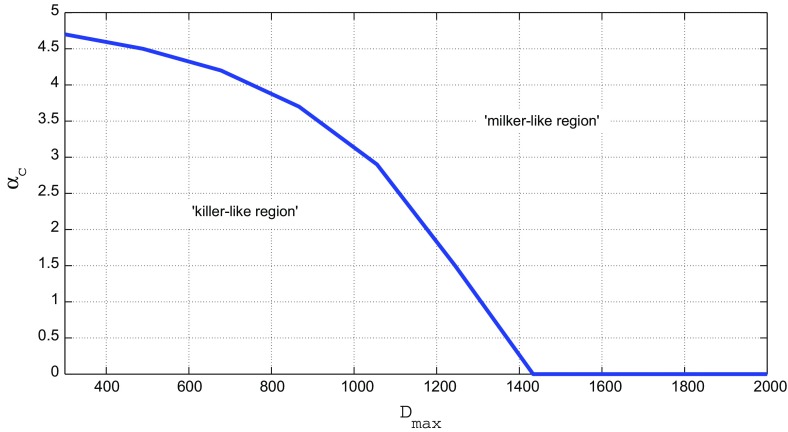
Behaviour of the critical value $\alpha _{c}$ as a function of $D_{{max}}$. The curve divides the ($D_{{max}}$ , $\alpha$) plane into two regions. Below the curve the optimal strategy for the pathogen is the ‘killer-like’ behaviour, above the curve the ‘milker-like’ behaviour is optimal

However, for high values of $D_{{max}}$ ($D_{{max}} \in (1200{-}2000)$ days), the linear dependence on $\alpha$ is shown on a much smaller interval. $R_0^{{opt}}$ tends to increase fast to a high value of about 32 as soon as $\alpha$ exceeds 1, and increases from there on only very slowly as a function of $\alpha$. In the limiting case of $D_{{max}} \rightarrow \infty$, $R_0^{{opt}}$ immediately attains the value of 32 as soon as $\alpha > 0$, and shows a linear dependence on $\alpha$ for all $\alpha$, but with a very small slope.

When observing the results in Fig. 2, the question arises for which combination of pathogen replication rate $\rho$ and mutation rate $\mu$, *R*_0_ attains its maximum, and how this combination changes when host longevity $D_{{max}}$ and/or between hosts contact rate $\alpha$ vary. This question is answered via the information in Fig. 3.

When $D_{{max}} \le 1000$ days ($\approx$ 3 years), we observe in Fig. 3 two completely distinct $R_0^{{opt}}$ regions: first, a region centred at about $\rho = 3$ and $\delta = 10^{-3}$ (low replication and high diversity, the ‘milker-like’ region, and second, a region centred at about $\rho = 8$ and $\delta = 10^{-6}$ (high replication and intermediate diversity, the ‘killer-like’ region). Low contact rates $\alpha$ are associated with the killer-like region and high rates with the milker-like region. This is to be expected: if contacts are rare, the pathogen should optimize the transmission rate of the disease by increasing the viral load during contact, because this is its only option to survive. The viral load is high in the killer-like regime and low(er) in the milker-like regime. A striking observation is that the jump between the regions occurs at a critical value of $\alpha$, which we denote by $\alpha _c$. On its turn, this critical value depends on $D_{{max}}$. In Fig. 4 we show how $\alpha _c$ behaves as a function of $D_{{max}}$. The curve in Fig. 4 divides this plane into two regions with completely distinct pathogen behaviour. Above the curve the ’milker-like’ behaviour (replication rate $\rho$ low and mutation rate $\delta$ high) is optimal for the pathogen and below the curve ’killer-like’ behaviour (replication rate $\rho$ high and mutation rate $\delta$ intermediate).

Another remarkable detail observed in Fig. 4 is that $\alpha _c$ vanishes if $D_{{max}}$ is bigger than some threshold, which occurs for our parameter values (taken from Lange and Ferguson 2009) at $D_{{max}} = 1425$ days. Above that threshold the killer-like region disappears and all optimum *R*_0_ values are attained in the milker-like region. This implies that for $D_{{max}}$ larger than this threshold the system always ultimately converges to approximately the same ’optimal’ values for $\rho$ and $\delta$, namely $\rho \approx 3$ and $\delta \approx 10^{-3}$. It should be noticed that this effect will only be seen if the epidemics gets enough time to adjust its parameters, since that is the philosophy underlying the introduction of $R_0^{{opt}}$ in (11). In this limiting case, $R_0^{{opt}}$ still increases linearly with $\alpha$, but quite slowly. It should be noted that the milker-like strategy is the one that eventually will show up if one waits long enough. In reality some diseases are still in the region of transient behaviour and thus on their way to a milker-like regime, but now still displaying killer-like behaviour.

## Discussion

A lot of studies relate pathogen success or failure to *R*_0_ by investigating how changes in the parameters governing model dynamics lead to changes in *R*_0_. The commonly considered factors, like force of infection, transmission probabilities and contact neighbourhoods are important. This certainly leads to more insight pertaining to the success or failure of infectious disease progression. Here, we have shown that the factor “remaining longevity of the host”, also influences the basic reproduction number of an epidemic and thus can essentially determine whether an outbreak is to be expected or not. We used a specific model from the literature to investigate the relation between *R*_0_ and remaining host longevity $D_{{max}}$. The within-host model used allows for variation in the diversity of the infectious agent, as well as for different rates of replication. Coupling it with the population-level model, we are able to show how the optimal strategy for the pathogen depends on the parameters. It should be noted that these optimal strategies will develop in the end and that during the transient phase suboptimal strategies could be observed. We found that optimal strategies depend heavily on $D_{{max}}$, as is clearly shown in Fig. 2. We conclude that the value of $D_{{max}}$ should be chosen in correspondence with the specific host-pathogen system under consideration. The value of $D_{{max}}$ may vary from very short, e.g., in the cases of fly or mosquito, to very long, e.g., for humans. One of the consequences of this insight is that the results given in Lange and Ferguson (2009), from which we adopted the modelling ideas, are valid for systems in which the hosts live at most 2 years after the start of the infection, because they fixed $D_{{max}}$ at that period, assuming that it would not change their conclusions. The present work shows that varying (remaining) host longevity can change conclusions drastically: in our long run simulations to determine the optimal *R*_0_ (Eq. 11), one of the three different regimes that Lange and Ferguson (2009) distinguished never pops up as the optimum strategy.

When expected host longevity is relatively short, we find that optimum between-host *R*_0_ only emerges in two clearly distinct regions in the plane spanned by the two relevant pathogen characteristics, namely $\delta$, ther probability of a mutation to be successful, and $\rho$, the replication rate. These two regions could be associated with the well-known ‘milker-like’ (low replication rate $\rho$) and ‘killer-like’ (high $\rho$) strategies. It should be noted that longevity may vary over sub-populations. For example, for humans longevity really hinges on the food availability in the environment and it ranges in WHO’s (2016) report between 50.1 and 83.7 years. This might also be the case for other species.

For given host longevity $D_{{max}}$, the choice between the milker-like and killer-like strategies depends on the contact rate $\alpha$. Figures 3 and 4 show that there exists a critical value $\alpha _c$ where the transition from killer-like to milker-like as the best strategy occurs. Figure 4 also shows that if $D_{{max}}$ exceeds some threshold value, the milker-like strategy is always optimal, independent of the contact rate $\alpha$. For the parameter setting used throughout this paper this threshold is approximately 4 years. This value itself is not the most important discovery, but the achieved insight is that such a threshold exists and can be quite easily calculated. If we combine the information contained in Figs. 3 and 4, we not only conclude that for $D_{{max}}$ above this threshold the milker-like strategy is optimal, but also that *R*_0_ in this regime linearly increases with the contact rate $\alpha$, although the slope of this increase is very small.

Our analysis suggests that in long-living hosts like humans most infectious diseases will finally attain a milker-like strategy. That does not imply that all human diseases are already of the milker-like type, but diseases that are already for a long time among us tend to be of milker-like type. Counterexamples are the diseases, so-called zoonotic diseases, that jump from animals to the human population. Examples like bird influenza and Ebola show that diseases that come from organisms with a shorter longevity. For example, bird influenza (de Jong et al. 2009) and fruit bats (Ebola, WHO 2017) are highly virulent (killer-like type).

It should be noted that both an individual based model or a physiologically structured population model incorporating a contact network, the within-host dynamics of the pathogen, and the stochastic longevity of hosts, might be able to show the impact of host longevity on *R*_0_ in a more rigorous way. However, this kind of modellling is beyond the scope of the present analysis.

The presented results show that host longevity really matters when pathogen fitness is investigated. When using *R*_0_ as a measure of fitness, $D_{{max}}$ should be taken equal to the expected remaining host longevity. The optimum infection strategies can only be reliably predicted if one uses appropriate estimates for host longevity.

## Supporting Information

*Appendix A* This appendix gives some typical results obtained through numerical simulations. We show typical types of within-host behaviour, as well as the fitness landscapes that result from the combination of the two models.

*Appendix B* In this section of the supporting documentation, we show how the within- and between-host models, given in the Methods section, have been made dimensionless and how they have been solved numerically.

## Electronic supplementary material

Below is the link to the electronic supplementary material.

Supplementary material 1 (pdf 560 KB)
