# Supplementary material for: R0: Host Longevity Matters
Source: Acta Biotheor. 2018 Feb 19;66(1):1–16. doi: 10.1007/s10441-018-9315-1 (PMC5874282; doi:10.1007/s10441-018-9315-1)
Supplement: Supplementary file 1 — Supplementary material 1 (pdf 560 KB) [file 10441_2018_9315_MOESM1_ESM.pdf]

R_0: Host Longevity Matters (Supplementary Material)

L.M. Viljoen[1,*], L. Hemerik [2], J. Molenaar[2],

1 Department of Mathematics and Applied Mathematics, North West University, Potchefstroom, North West, South Africa
2 Biometris, Department of Mathematical and Statistical Methods, Wageningen University, Wageningen, The Netherlands

*** thinus.viljoen.tv@gmail.com**

Abstract

In this supplementary material we present in Appendix A some typical results from numerical simulations and in Appendix B the dimensionless within- and between-host models used to derive these results and the way they are solved numerically.

Appendix A

Here, we present two types of model outcomes from numerical simulations: (i) using the within-host system to show how different pathogen space parameters influence the dynamics of this system and (ii) linking the within-host behaviour to between-host dynamics, using this concurrent implementation to calculate R_0.

As discussed in the main text (R_0 : Host Longevity Matters) the successful dynamics of a pathogen is well defined in terms of R_0, which depends on various parameters, both within- and between-host. For the purpose of this study we consider only those parameters that have an essential impact on R_0: parameters ρ (replication rate) and δ (mutation rate) that pathogens can control when developing strategies to ensure immune evasion (together referred to as 'pathogen space'), and parameters α (contact rate between hosts) and, of course, D_{max} (host longevity), since that is the core parameter in our analysis.

The longevity of a host, D_{max}, is an essential host-specific factor that has a noticeable influence on R_0. Unless stated otherwise, default parameter choices were incorporated during numerical simulations, given in Tables 1 and 2 of the main text.

Within-host dynamics

As for the within-host model, given in equations (1-7) in the main text, Fig. A1 shows typical examples of how different pathogen space parameter combinations influence within-host dynamics. Three distinct types of behaviour emerge, as already stated by Lange and Ferguson (2009)f, yielding different model outcomes in terms of duration of infection, average strain numbers, and levels of infectiousness:

(1) The first type of behaviour (type I) is characterised by more stochastic durations of infections. This occurs when the replication rate is very high, at intermediate levels of diversity. Typical infections that correspond with this behaviour are flu-like infections.

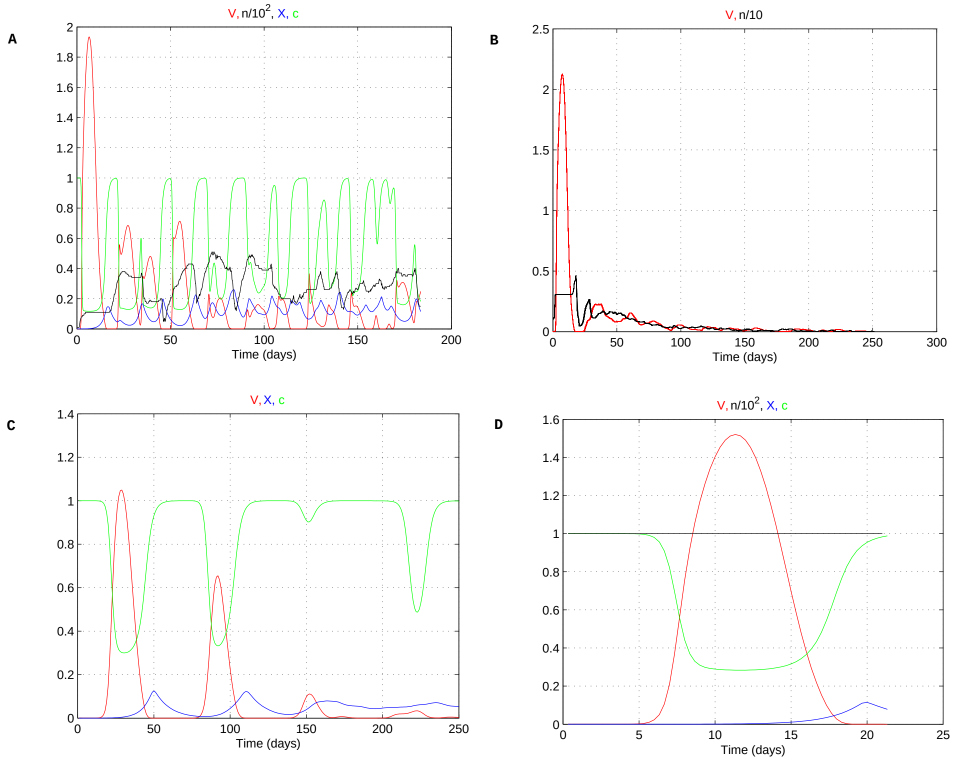

Figure A1: Within-host dynamics for variations in pathogen replication rate ρ and diversity parameter δ. Three types of within-host behaviour is observed. [A] One realization for high $\rho = 8$ and intermediate $\delta = 10^{-6}$ (type I). Durations of infection vary greatly for type I, reaching from a few days, up to D_{max}. Due to the stochasticity observed in type I dynamics, average dynamics is considered. [B] Average type I behaviour over 100 realizations. [C] Average type II behaviour is characterized by long-lived infections (almost always) produced by low $\rho = 3$ and high $\delta = 10^{-3}$. [D] For pathogens that replicate slowly and that are not very diverse ($\rho = 3$ and $\delta = 10^{-9}$) infections end after only a few days, with the initial strain being dominant and a small probability of mutations occurring.

(2) When within-host pathogen replication is low and diversity is high, the infectious period lasts very long and we notice very little stochastic behaviour, typical of infections such as HIV. We notice low average pathogen loads and low strain numbers at large times, with the initial part of the infectious period dominating overall behaviour for this type II infections.

(3) Finally, when replication is slow and there is little diversity of the infectious agent, we notice short lived infections and low average strain numbers (type III). This behaviour is comparable to childhood like infections, such as measles.

Between-host dynamics

Here, we consider what will happen if we combine the different within-host dynamics, identified in the previous section, with between-host dynamics, given in equations (8-12) in the main text. This is done via fitness landscapes that reflect how duration of infection, cumulative pathogen load, and R_0 behave as functions of the within-host parameters ρ and δ.

Fig. A2 gives typical average behaviour of the firness landscape for an intermediate

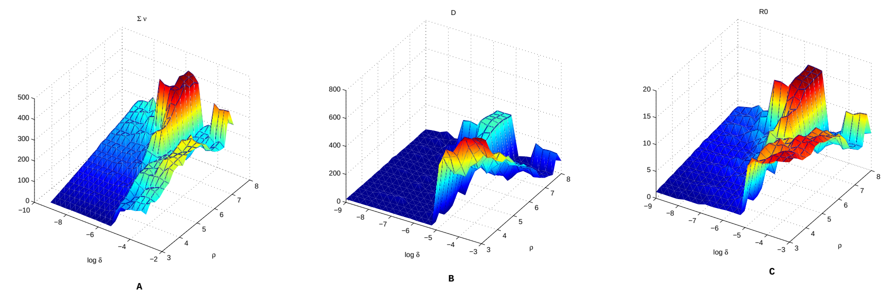

Figure A2: Average landscapes reflecting model outcomes. [A] Cumulative pathogen load, [B] Duration of infection and [C] R_0, for variations in pathogen space parameters ρ and δ. These results are obtained with $\alpha = 5$.

value of $\alpha = 5$. Cumulative pathogen load is shown in panel A; the landscape peaks at high levels of pathogen replication and intermediate levels of diversity, corresponding with one of the regions where optimum R_0 occurs.

In panel B of Fig. A2 we see that the average duration of an infection is characterised by endemic behaviour at low levels of replication and high levels of diversity. Panel C of Fig. A2 shows the two regions in pathogen space where optimum between-host R_0 is possible. When host longevity is more and more increased, the R_0 landscape more ande more resembles the duration landscape in Panel A of Fig. A2 and optimum R_0 is only achieved by pathogens that replicate slowly.

Appendix B

In this appendix we show how the within- and between-host models, given in equations (1 - 12) in the main text, may be written in dimensionless form. We also describe how the between-host dimensionless equation is numerically solved by a process of discretization and numerical approximation. Default parameter values are given in Tables 1 and 2 of the main text.

Within-host model

The within-host model equations (1-7) in the main text are dimensionful. The consequence is that this system of ordinary differential equations yields state variables that may vary greatly in magnitude. E.g., total pathogen load may reach levels of $\nu \approx 10^{11}$, whereas adaptive immunity saturates at $\eta = 10^5$. These huge differences may lead to numerical instabilities and inaccurate results of simulations. That is the reason why we have put all models in dimensionless form, since this leads to state variables and parameters having order of magnitude one. With this approach we also overcome possible problems caused by time scale differences when within- and between-host dynamics are coupled. Non-dimensionalization is not a unique procedure (Groesen and Molenaar 2007). In the present case it suffices to apply a straightforward scaling procedure:

$$V_i^* = \frac{V_i}{\varpi},\ X_i^* = \frac{X_i}{\eta},\ C^* = \frac{C}{C_0}\ \text{, and } t^* = \rho\, t, \tag{B1}$$

where ϖ is the maximum obtained for the viral strain loads. Below we derive a reasonable value for ϖ. The parameter η is chosen to scale the X_i's, as this parameter denotes the critical load above which immunity saturates. C_0 is the maximum number of resource cells and therefore a logical choice for scaling C. Time is scaled using the parameter ρ. This enables us to reduce the number of parameters by 1.

Substituting (B1) into equations (1,2,3) in the main text, we obtain:

$$\frac{dV_i^*}{dt^*} = (1-\mu)\,\frac{\nu_1\, C_0}{\varpi}\, V_i^*\, \frac{C^*}{\nu^* + \frac{\nu_1\, C_0}{\varpi}\, C^*} - \frac{\psi}{\rho}\, V_i^* - \frac{\sigma\,\eta}{\rho}\, V_i^*\, \Sigma_{k=1}^n\, y(\varrho_{ik})\, X_k^*\ , \tag{B2}$$

$$\frac{dX_i^*}{dt^*} = \frac{\xi}{\rho}\,(x_0^* - X_i^*) + \frac{\zeta}{\rho}\, X_i^*\, \frac{V_i^*}{V_i^* + \frac{\eta}{\varpi}}\ , \tag{B3}$$

$$\frac{dC^*}{dt^*} = \frac{\kappa}{\rho}\,(1 - C^*) - \Sigma_{i=1}^n V_i^*\, \frac{C^*}{\nu^* + \frac{\nu_1\, C_0}{\varpi}\, C^*}\ . \tag{B4}$$

The within-host model now has the dimensionless parameters μ, $\frac{\nu_1\, C_0}{\varpi}$, $\frac{\psi}{\rho}$, $\frac{\sigma\,\eta}{\rho}$ $\frac{\xi}{\rho}$, $\frac{\zeta}{\rho}$, $\frac{\eta}{\varpi}$ and $\frac{\kappa}{\rho}$. For the integration of this set of $2n+1$ differential equations we use the *ode45*-routine in Matlab that utilises a fourth-fifth-order Runge-Kutta method. The initial values for the state variables are set to the (scaled) values, with $V_0(0) = \frac{\nu_0}{\varpi}$, $X_0(0) = \frac{x_0}{\eta}$ and $C(0) = 1$.

Between-host model

The between-host model is described in equations (4-8) in the main text with as core the integro-differential equation (8). We put (8) in dimensionless form by scaling the susceptible population via $Z = \frac{S}{N}$ and the time variable via $t^* = t\rho$, which is consistent with the scaling of the time in the within-host model. Substituting these scalings in (8) we find:

$$\begin{aligned}\frac{dZ}{dt^*} = {} & p_6(1-Z) - \\ & q^* Z - p_6 p_7 Z \int_0^{t^*} q^*(\tau)(1 - Z(t^* - \tau))d\tau + \\ & p_7 Z \int_0^{t^*} q^*(\tau) \frac{dZ}{dt^*}(t^* - \tau)d\tau \end{aligned} \tag{B5}$$

where $p_6 \equiv \frac{\omega}{\rho}$, and $p_7 \equiv \phi\, N$.

The transmission rate q defined in (4) in the main text has dimension T^{-1}. By setting $\beta^* = \alpha^*\gamma/N$, with $\alpha^* = \alpha/\rho$, we get the dimensionless form $q^* = \beta^*(1 - \exp(-\nu^*/\nu_{\mathrm{T}}^*))$. Here, ν^* is the total pathogen load from the within-host system and $\nu_T^* = \nu_T/\varpi$ represents the (scaled) infectiousness threshold.

To numerically solve the dimensionless integro-differential equation (B5), we use a forward-difference technique to approximate the derivatives, and apply the trapezium rule to approximate the integral terms. This leads to:

$$\frac{Z_{i+1} - Z_i}{\Delta t} = p_6(1 - Z_i) - q_i^* Z_i -$$
$$p_6\, p_7 \left[\frac{\Delta t}{2} \sum_{j=0}^{i} \epsilon(j)\, q_j^* \,(1 - Z_{i-j}) \right] Z_i +$$
$$p_7 \left[\frac{\Delta t}{2} \sum_{j=0}^{i} \epsilon(j)\, q_j^* \, \left(\frac{Z_{i-j+1} - Z_{i-j}}{\Delta} \right) \right] Z_i, \qquad \text{(B6)}$$

where Δt is the time step, and $\epsilon(j) = 1,$ if $j = 0$ or $j = i$, and $\epsilon(j) = 2$, otherwise.

From algebraic manipulation, we may rewrite (B6) such that Z_{i+1} is defined explicitly in terms of Z_i and the other variables and parameters in the model:

$$Z_{i+1} = \frac{1}{1 - \frac{1}{2}\Delta t\, p_7\, q_0\, Z_i} \Bigg\{$$
$$Z_i + \Delta t\, p_6\,(1 - Z_i) - \Delta t\, q_i^* Z_i -$$
$$(\Delta t)^2 \left(\frac{p_6\, p_7}{2} \right) \left[\sum_{j=0}^{i} \epsilon(j)\, q_j^* \,(1 - Z_{i-j}) \right] Z_i +$$
$$\frac{1}{2} \Delta t\, p_7 Z_i \left[q_0^*\, Z_i - \sum_{j=1}^{i} \epsilon(j)\, q_j^* \,(Z_{i-j+1} - Z_{i-j}) \right] \Bigg\}$$

We start the evaluation of this recurrence relation with $Z_0 = \frac{N-1}{N}$. In this way we are able to explicitly calculate the number of susceptible hosts when time runs.

References

Lange, A. & Ferguson, N.M. (2009) Antigenic Diversity, Transmission Mechanisms, and the Evolution of Pathogens PLOS Computational Biology, October 2009.
Van Groesen, E,, Molenaar, J. (2007) Continuum Modeling in the Physical Sciences. SIAM, pp. 228, ISBN 978-0-898716-25-2.
